# Is More Perceived Empathy Better? Large Language Models, Communication, and Safety in Health Care

**DOI:** 10.2196/113223

**Published:** 2026-11-10

**Authors:** Rebecca Grochow Mishuris, Sara G Murray, Deepti Pandita

**Affiliations:** 1 Mass General Brigham, 399 Revolution Drive, Somerville, MA, 02145, United States, 1 617-732-6040; 2 Johns Hopkins Medicine, Baltimore, MD, United States; 3 University of California, Irvine, Irvine, CA, United States

**Keywords:** patient-clinician communication, generative AI, patient portal, large language model, physician-patient communication, empathy, artificial intelligence in health care

## Abstract

Richter and colleagues offer a careful systematic review of how large language model–based tools affect physician-patient communication, but the outcomes that matter most in deciding to scale a solution—trust and safety—are still unknown. Before deploying these tools, health systems need local validation, ongoing monitoring for accuracy and equity, and pragmatic trials that measure trust, safety, and outcomes alongside perceived empathy.

Is more perceived empathy better? Richter and colleagues, in their systematic review and meta-analysis of large language model (LLM)–based tools in physician-patient communication [1], report that across 10 studies, AI-generated responses were rated more empathetic than physicians’, with a large pooled effect (standardized mean difference 1.02, 95% CI 0.44‐1.60) and supporting odds ratio of 5.5. AI answers were also longer, clearer, and often rated more useful. Only one disease-specific study found no difference in empathy. The authors are appropriately measured in their conclusion: no randomized trials, no longitudinal data, no direct measurement of trust, and substantial heterogeneity (*I*²=73%) [1]. That heterogeneity likely reflects differences in study design, response format, raters, and prompting as much as underlying communicative quality, highlighting the importance of validation in specific workflows and populations where tools will be deployed. The result echoes the primary studies that first drew attention to “artificial empathy” [2] and arrives as generative AI diffuses through health care faster than our capacity to evaluate it [3].

We take the empathy finding seriously, but what concerns us more is what it leaves out, along with one important safety caveat. The review measures perceived empathy in blinded ratings of written answers, not communication as it actually unfolds in a clinical relationship: correcting misunderstandings, clarifying ambiguity, responding to emotion, and adapting as new information emerges. In routine care, empathy is not only tone; it is timing, calibration, and response to evolving patient cues. Empathy is also a proxy for outcomes health systems are accountable for: accuracy, comprehension, equity, trust, adherence, and safety. Written, decontextualized ratings have limited pragmatic validity because they strip away dialogue and the chance for iterative meaning-making with a patient over time. The review’s most consequential observation is that in one study, reviewers flagged some AI answers as potentially harmful, yet patients rated those same answers highly and could not tell them apart [1,4]. These answers can sound convincing even when inaccurate, and that should shape deployment policy more than the effect size does.

This is why “human in the loop” cannot be a disclaimer; it has to be a design and governance choice. The operative questions are not whether an LLM can sound empathetic, but how reliance, authority, and oversight are structured in real workflows, when the tool is advisory versus definitive, and which roles may rely on it. Direct oversight mitigates risk only if the clinician has the knowledge, time, tooling, and incentive to catch what the patient cannot. Otherwise, oversight becomes a rubber stamp, one more alert that busy clinicians learn to override.

There is also a gap between what was studied and what is already being deployed. The review’s interventions are simulated, asynchronous, and text-based. In contrast, tools are already in production (eg, draft portal message replies and ambient documentation) ahead of the evidence base the authors call for, with real-world evaluations only now emerging [5]. Closing that gap responsibly means matching evaluation rigor to use-case risk. A clinician-edited draft message is different clinically, legally, and ethically from an unsupervised patient-facing chatbot. The two should not clear the same bar.

A model that generalizes empathetic language across patients, clinicians, and third-party raters has demonstrated a real capability, but that capability alone does not make a tool ready to deploy. Readiness requires local validation against patients and message types before go-live; longitudinal audits for hallucination, clinical inconsistency, and drift; readability testing that includes low–health-literacy cohorts; and subgroup-level review to identify whether inaccuracies cluster by condition, language, age, disability or assistive needs, prior knowledge, or question type. It also requires workflow monitoring: does the tool reduce clinician burden, or does it create new review work, automation bias, or uneven benefit? The review notes that AI text tends to be longer and can exceed the reading level that patients with limited health literacy require [1]. A tool that improves average satisfaction while widening comprehension gaps is not a success; it is a new disparity. Because evaluation capacity is unevenly distributed, health systems will need shared evaluation methods and local capacity so tools meant to democratize communication do not deepen the digital divide [6].

Trust shapes whether patients attend to advice, ask follow-up questions, and adhere to plans, especially when AI assistance may be invisible in the care relationship. No included study measured trust directly, yet believing AI was involved can change how patients judge medical advice [1]. Trust should be assessed with validated patient-reported measures, including trust-in-technology or trust-in-health care instruments adapted to AI-assisted communication, and triangulated with behavioral proxies such as follow-up question rates, confirmation-seeking, adherence indicators where available, and escalation or care-seeking after AI-drafted guidance. Disclosure should therefore be treated as policy, not etiquette. Whether and how AI assistance is disclosed, and how disclosure changes trust over time, should be codified and studied rather than assumed. The review is best read as a specification for the evidence we lack (Table 1): pragmatic and randomized trials that evaluate AI-supported versus standard communication and measure understanding, trust, adherence, safety, and outcomes—not perceived empathy alone. We also need longitudinal follow-up to know whether machine empathy has durability, reported against shared priorities for trustworthy health AI [7,8]. This must be balanced against the rapid growth of the technology and the opportunities for health improvement that come with it.

The authors ask whether these tools will prove a “fountain” or a “Pandora’s box” [1]. Governance will determine which path they take. Richter and colleagues have shown that LLMs can communicate with warmth and clarity. The harder question is whether they can do so safely and durably at scale, which depends on validation, monitoring, disclosure, oversight, and capacity sufficient to reach the patients and systems most at risk of being left out. The empathy finding is real. The safety caveat matters more for how quickly, and how carefully, health systems choose to deploy these tools.

**Table 1.** From gaps in the evidence to health-system action.

What Richter et al [1] established	What remains unknown	Action for health-system AI leaders within the context of the unknown
LLM^a^ replies rated more empathetic than physicians	Whether perceived empathy from an LLM can lead to durable trust	Leverage LLM-drafted empathy in helping draft communication with human oversight
Higher clarity, completeness, and usefulness ratings for LLM replies	Comprehension for low–health-literacy and non–English-speaking patients	Add reading level, language, and readability to acceptance testing. Leverage tools to distill information for diversity of reading level, health literacy, and other languages.
Patients could not detect potentially harmful answers	Real-world harm rates under real-life conditions	Design oversight into the workflow; monitor edit rates and automation bias
No trust outcome measured directly	Whether AI empathy builds or erodes trust, especially after disclosure	Set disclosure as policy, particularly where human oversight is not guaranteed; name trust as a monitored metric
No randomized controlled trials, longitudinal data, or real-world data	Effects on adherence, safety, and outcomes over time	Sponsor pragmatic/randomized local trials; require standardized monitoring
Evaluation and monitoring assumed, not resourced	How underresourced systems keep pace safely	Invest in shared evaluation and local capacity to avoid a widening divide

a LLM: large language model.

## Abbreviations

LLM: large language model
